# Comparison of RECIST 1.1 and iRECIST in Patients Treated with Immune Checkpoint Inhibitors: A Systematic Review and Meta-Analysis

**DOI:** 10.3390/cancers13010120

**Published:** 2021-01-01

**Authors:** Hyo Jung Park, Gun Ha Kim, Kyung Won Kim, Choong Wook Lee, Shinkyo Yoon, Young Kwang Chae, Sree Harsha Tirumani, Nikhil H. Ramaiya

**Affiliations:** 1 Department of Radiology and Research Institute of Radiology, University of Ulsan College of Medicine, Asan Medical Center, Seoul 05505, Korea; happyeahj@gmail.com (H.J.P.); idgunkim@gmail.com (G.H.K.); cwlee@amc.seoul.kr (C.W.L.); 2 Department of Oncology, University of Ulsan College of Medicine, Asan Medical Center, Seoul 05505, Korea; shinkyoyoon82@gmail.com; 3 Department of Medicine, Feinberg School of Medicine, Robert H. Lurie Comprehensive Cancer Center, Northwestern University, Chicago, IL 60611, USA; young.chae@northwestern.edu; 4 Department of Radiology, University Hospitals Cleveland Medical Center, Case Western Reserve University, 11100 Euclid Ave, Cleveland, OH 44106, USA; sreeharsha.tirumani@uhhospitals.org (S.H.T.); Nikhil.Ramaiya@uhhospitals.org (N.H.R.)

**Keywords:** RECIST, iRECIST, immunotherapy, checkpoint inhibitor, treatment efficacy

## Abstract

**Simple Summary:**

It is controversial whether iRECIST has a significant impact over RECIST 1.1 in evaluating the efficacy of immune checkpoint inhibitor treatment. We aimed to evaluate the impact of iRECIST on assessing treatment efficacy of immune checkpoint inhibitors over RECIST 1.1 through a systematic review and meta-analysis. Compared to RECIST 1.1, iRECIST had no impact on the overall response rate and disease control rate but detected 3.9% of patients with discordance in the date of progressive disease determination due to pseudoprogression and prolonged restricted mean progression-free survival time by 0.46 months. Therefore, the application of iRECIST had no impact on the response-related endpoints but had a minor impact on the survival endpoint, compared to RECIST 1.1. Such a modest benefit of iRECIST should be considered when we design a clinical trial for immune checkpoint inhibitors.

**Abstract:**

Despite wide recognition of iRECIST, evidence regarding the impact of iRECIST over RECIST 1.1 is lacking. We aimed to evaluate the impact of iRECIST on assessing treatment efficacy of immune checkpoint inhibitors (ICIs) over RECIST 1.1. Articles that evaluated the treatment response and outcome based on both RECIST 1.1 and iRECIST were eligible. Data regarding overall response rates (ORR) and disease control rate (DCR) based on RECIST 1.1 and iRECIST, and data required to estimate individual patient data of progression-free survival (PFS) were extracted. Estimates were compared using meta-regression and pooled incidence rate ratios. The pooled difference of restricted mean survival time (RMST) of PFS between two criteria were calculated. Eleven studies with 6210 patients were analyzed. The application of iRECIST had no impact on the response-related endpoint by showing no significantly different ORR and DCR from RECIST 1.1 (pooled ORR, 23.6% and 24.7% [*p* = 0.72]; pooled DCR, 45.3% and 48.7% [*p* = 0.56] for iRECIST and RECIST 1.1, respectively) and had a minor impact on a survival endpoint by showing longer RMST of PFS than RECIST 1.1 (pooled difference, 0.46 months; 95% CI, 0.10–0.82 months; *p* = 0.01). Such a modest benefit of iRECIST should be considered when we design a clinical trial for immune checkpoint inhibitors.

## 1. Introduction

Cancer immunotherapy introduced revolutionary changes in the treatment of several cancers. Immune checkpoint inhibitors (ICIs) targeting programmed cell death protein 1 (PD-1), its ligand (PD-L1), or cytotoxic T-lymphocyte-associated antigen 4 (CTLA-4) are among the mostly used therapeutics in various cancers in clinical trials and practice [1,2]. New response patterns have been observed in patients treated with ICIs, including an increase in tumor burden followed by a tumor response, a phenomenon termed “pseudoprogression” [3,4,5,6,7]. Response evaluation criteria in solid tumors (RECIST) 1.1, the current standard response evaluation criteria [8], may not capture the pseudoprogression as a treatment effect, but rather classify it as progressive disease (PD) and lead to treatment discontinuation.

Efforts have been made to develop reliable response evaluation criteria to account for pseudoprogression during ICI treatment [6,7,9]. Immune-related response criteria (irRC), developed based on World Health Organization criteria in 2009, allows for response evaluation after initial PD [7], and additional adaptation to the RECIST scheme was made with the immune-related RECIST (irRECIST) developed in 2013 [6,10]. However, criticisms have been raised regarding irRC and irRECIST as PD is confirmed in cases showing a stable or only minor tumor size decrease following pseudoprogression. Also, various modifications of irRC and irRECIST have been adopted depending on the clinical trial protocols, leading to inconsistencies across studies [11]. To address these issues, immune RECIST (iRECIST) was proposed by the official RECIST working group in 2017 [9] and has been widely adopted in clinical trials.

In most clinical trials, iRECIST is used for exploratory endpoints for treatment efficacy evaluation, and RECIST 1.1 is used to assess the primary endpoints, which may result in variability in data interpretation [1]. Besides, iRECIST may increase the burden in image interpretation and data management. Nevertheless, many clinical trials use both RECIST 1.1 and iRECIST at the same time to capture pseudoprogression to obtain a rationale to continue treatment after initial PD on RECIST 1.1, which is called treatment beyond progression.

Despite wide recognition of iRECIST, it is still controversial whether iRECIST has a significant impact over RECIST 1.1 on evaluating the efficacy of ICI treatment. Currently, there have been multiple scattered individual studies to suggest data on the efficacy endpoints per RECIST 1.1 and iRECIST, and no attempt has yet been made to generate a systematic summary about the impact of iRECIST on efficacy endpoints, which would be of great help for a more evidence-based standardized management of patients treated with ICIs. To this end, we performed this systematic review and meta-analysis to provide evidence-based insight regarding the impact of iRECIST on assessing the treatment efficacy of ICIs compared with RECIST 1.1.

## 2. Results

### 2.1. Study Characteristics

**Figure 1:**
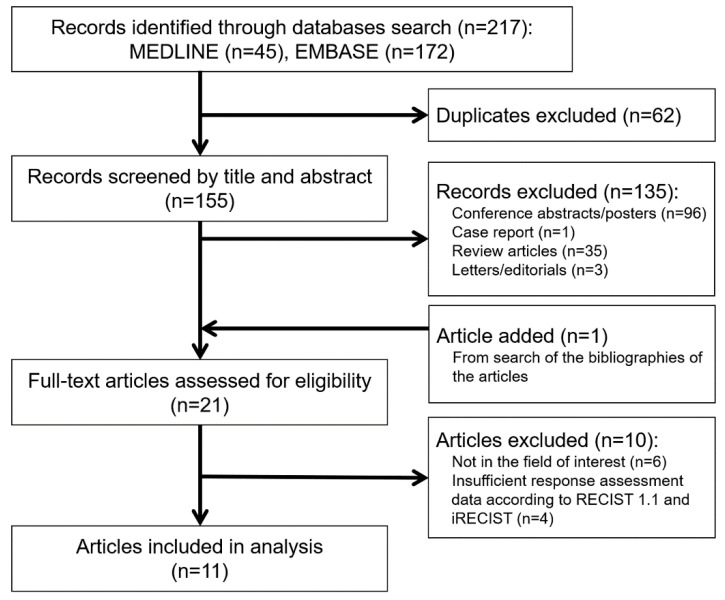
Flow diagram of the study selection process.

The study search process is shown in Figure 1. The characteristics of the 11 studies [12,13,14,15,16,17,18,19,20,21,22] including 6210 patients are summarized in Table 1. One study was a pooled analysis of individual patient data (IPD) from 14 Food and Drug Administration (FDA)-approved randomized controlld trials (RCTs), one was an RCT, one was a phase II clinical trial, and eight were observational studies (1 prospective study and 7 retrospective studies). Regarding the tumor type, five studies included non-small cell lung cancer, two included breast cancer, one included melanoma, one included urothelial carcinoma, and two included multiple types of cancer (≥3). Most patients (73.7–100.0%) were pretreated with systemic therapy in seven studies. In one study, 21.6% of patients were pretreated with systemic therapy.

Regarding the quality of the studies, the Cochrane Risk of Bias 2.0 tool showed a low risk of bias for one RCT (Figure S1), and the Newcastle-Ottawa Scale scores for each of the nine non-randomized studies ranging from 6 to 8 points (Table S1), indicating the high quality of included studies.

### 2.2. Comparison of Endpoints between RECIST 1.1 and iRECIST

#### 2.2.1. Response-Related Endpoints

**Figure 2:**
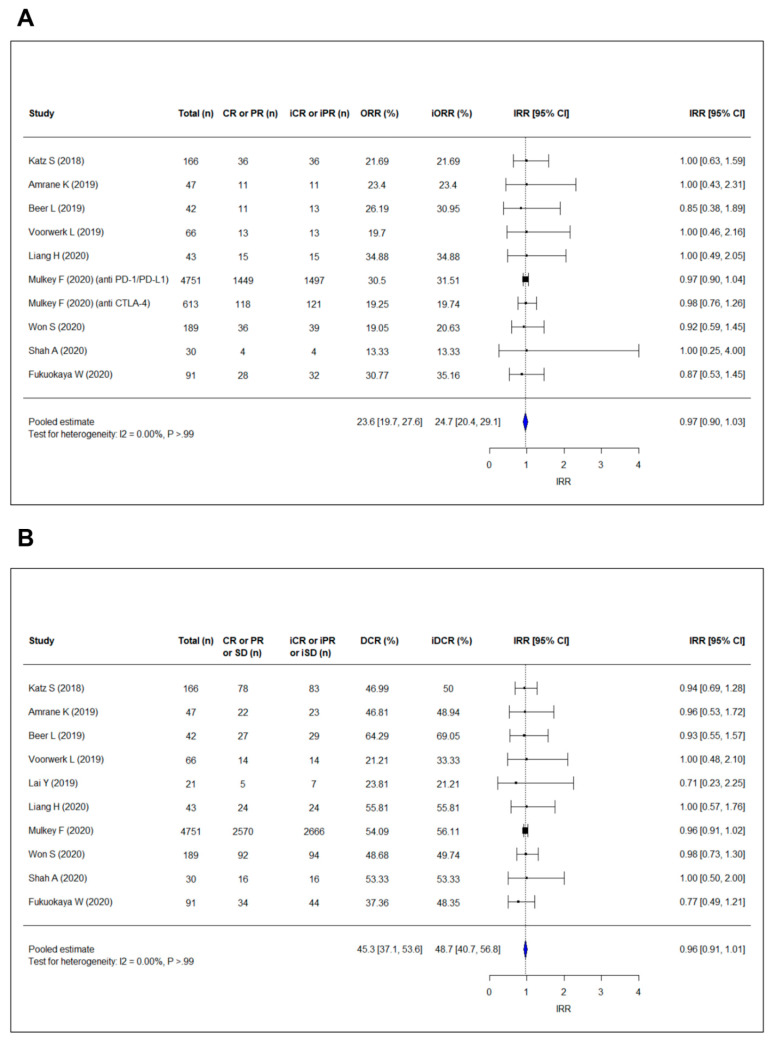
Forest plots showing the pooled estimate of (**A**) incidence rate ratio of ORR and (**B**) incidence rate ratio of DCR according to RECIST 1.1 and iRECIST. The pooled incidence rate ratio of ORR per RECIST 1.1 and iORR per iRECIST is 0.97 (95% CI, 0.90–1.03), and the pooled incidence rate ratio of DCR per RECIST 1.1 and iDCR per iRECIST is 0.96 (95% CI, 0.91–1.01), indicating no significant increase in both ORR and DCR using iRECIST compared with RECIST 1.1. “i” indicates immune responses assigned using iRECIST. Abbreviations: CI, confidence interval; CR, complete response; DCR, discase control rate; IRR, incidence rate ratio; ORR, overall response rate; PR, partial response; SD, stable disease.

The overall response rates per RECIST 1.1 (ORRs) of included studies ranged from 13.3% to 34.9% and the ORRs per iRECIST (iORRs) ranged from 13.3% to 35.2% (Figure 2A). The pooled ORR and iORR were 23.6% (95% confidence interval [CI], 19.7–27.6%) and 24.7% (95% CI, 20.4–29.1%), respectively (Figure S2). Meta-regression confirmed that there was no significant difference between the pooled ORR and iORR (*p* = 0.72). The pooled incidence rate ratio between ORR and iORR was 0.97 (95% CI, 0.90–1.03), also indicating no significant difference between ORR and iORR. No heterogeneity was present (I^2^ = 0.00%; *p* > 0.99).

As presented in Figure 2B, disease control rates per RECIST 1.1 (DCRs) ranged from 21.2% to 64.3%, and the DCRs per iRECIST (iDCRs) ranged from 21.2% to 69.0%. The pooled DCR and iDCR were 45.3% (95% CI, 37.1–53.6%) and 48.7% (95% CI, 40.7–56.8%), respectively (Figure S3). There was no significant difference between DCR and iDCR (meta-regression, *p* = 0.56; pooled incidence rate ratio, 0.96 [95% CI, 0.91–1.01]). Heterogeneity was absent (I^2^ = 0.00%; *p* > 0.99). In these meta-analyses, no significant publication bias was detected on the funnel plots and the rank correlation test (Figure S4).

Table 2 lists the pooled incidence of response-related endpoints in the subgroups classified according to the tumor type, drug type, study design, and prior systemic treatment. All sensitivity analyses showed no significant difference in estimates between the pooled ORR and iORR (*p* ≥ 0.63), and between the pooled DCR and iDCR (*p* ≥ 0.23). The pooled rate of PD date discordance between RECIST 1.1 and iRECIST were equal or less than 5.4%. 

#### 2.2.2. Survival Endpoints

**Figure 3:**
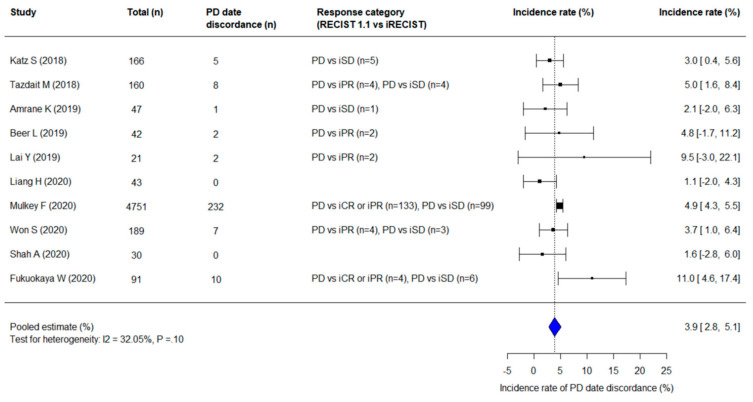
A forest plot showing the pooled incidence rate of PD date discordance between RECIST 1.1 and iRECIST. The pooled incidence rate of PD date discordance between RECIST 1.1 and iRECIST was 3.9%; 95% CI, 2.8–5.1%). “i” indicates immune responses assigned using iRECIST. Abbreviation: CR, complete response; PD, progressive disease; PR, partial response; SD, stable disease.

The pooled incidence rate for PD date discordance between RECIST 1.1 and iRECIST was 3.9% (95% CI, 2.8–5.1%) (Figure 3). There was no significant heterogeneity (I^2^ = 32.15%; *p* = 0.10) and no significant publication bias (*p* = 0.73). The discordant cases showed PD on RECIST 1.1 was followed by tumor shrinkage; they were reset as iSD, iPR, or iCR upon subsequent assessment based on iRECIST, with “i” indicates immune responses assigned using iRECIST.

**Figure 4:**
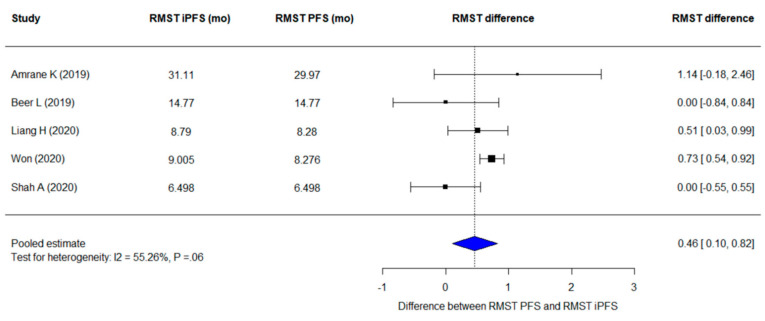
A forest plot for the difference of RMST of PFS per RECIST 1.1 and iPFS per iRECIST. The pooled difference of RMST of PFS per the two criteria are 0.46 (95% CI, 0.10–0.82 months; *p* = 0.01). iRECIST-based estimates are indicated as “i”. Abbreviations: PFS, progression-free survival; RMST, restricted mean survival time.

The pooled difference between restricted mean survival time (RMST) per iRECIST (RMST_iPFS_) and that per RECIST 1.1 (RMST_PFS_) was 0.46 months (95% CI, 0.10–0.82 months; *p* = 0.01), implying that the pooled mean progression-free survival per iRECIST per iRECIST (iPFS) was slightly but significantly longer than the pooled mean PFS per RECIST 1.1 (Figure 4). Heterogeneity was found in pooling the RMST difference (I² =55.3%; *p* = 0.06). There was no significant publication bias (*p* = 0.68) as well as the lack of asymmetry in funnel plots (Figure S4).

## 3. Discussion

In this meta-analysis, the response-related endpoints of ORR and DCR did not differ significantly between RECIST 1.1 and iRECIST, while the survival endpoint differed significantly. The pooled incidence rate of PD date discordance between RECIST 1.1 and iRECIST was 3.9%, and the pooled RMST of PFS was longer with iRECIST than that with RECIST 1.1 by 0.46 months.

There were only slight differences between the pooled ORR and iORR (23.6% vs. 24.7%) and between the pooled DCR and iDCR (45.3% vs. 48.7%) mainly because RECIST 1.1 and iRECIST have the same definitions of complete response, partial response, and stable disease [1]. Those differences can be explained by that some patients with initial PD on RECIST 1.1 were reset to iCR, iPR, or iSD on subsequent imaging due to immunotherapy-related pseudoprogression [9]. Likewise, the pooled incidence rate of PD date discordance (3.9%) between RECIST 1.1 and iRECIST was associated with the major concept of iRECIST, “resetting the bar,” which occurs if RECIST 1.1 defined PD is followed by tumor shrinkage [9]. These results are in line with the recent data that pseudoprogression is a rare phenomenon in ICI treatment [1,23]. Studies demonstrated that patients showing PD during ICI treatment may show a prolonged or delayed response or durable disease stabilization and it may impact the clinical outcome [24]. Therefore, concerns may arise that the use of RECIST 1.1 would lead to premature discontinuation of possibly effective treatments in a subset of patients receiving ICI treatment [1]. In our study, the pooled difference between RMST_iPFS_ and RMST_PFS_ (0.46 months; equivalent to 14.0 days) implies that the overall mean duration of survival without disease progression was slightly longer with the iRECIST scheme than with the RECIST 1.1 scheme.

Although iRECIST captured the benefit of ICI treatment in a subset of patients which RECIST 1.1 did not, and significantly prolonged PFS compared to RECIST 1.1, the magnitude of benefit was modest (3.9% of patients with pseudoprogression and PFS prolongation by 0.46 months), and these results should be interpreted with caution concerning the benefit and drawbacks of iRECIST application in clinical trial and practice [20,23]. While the application of iRECIST is beneficial to fully investigate the atypical response such as pseudoprogression and treatment efficacy, it adds considerable burden in imaging interpretation, data management, statistical analysis, cost, and potential risk of maintaining futile treatment. Further studies are necessary to evaluate whether the impact of iRECIST on treatment efficacy demonstrated in our analysis can outweigh the increased burden.

The key differences between RECIST 1.1 and iRECIST are PD determination methods and treatment beyond progression. When PD occurs according to RECIST 1.1, iUPD is assigned according to iRECIST which requires confirmation on subsequent imaging at 4–8 weeks intervals. Thus, iRECIST allows treatment past RECIST 1.1-defined PD as long as the patient is clinically stable and tolerates the therapy [9]. In iRECIST, if the initial PD on RECIST 1.1 is not confirmed on subsequent imaging, but instead the tumor is decreased or stable, then the response is reset to the iCR, iPR, or iSD. Thus, iRECIST reflects the pseudoprogression well. However, controversy remains as the incidence of pseudoprogression is low, less than 10% [23] Treatment beyond progression might have risks of continuing futile treatment and delaying switch to other treatment options in patients with true progression. Although recent studies demonstrated that treatment beyond progression can provide the benefit of overall survival [20], further studies are necessary to evaluate the survival benefit of treatment beyond progression based on iRECIST.

In our meta-analysis, we included RCTs as well as observational studies. RCT is not always the optimal study design for evaluating the treatment effect [25,26], as in the present study. Although Mulkey and colleagues performed a pooled study using IPD of 14 RCTs [19], their study had a significant limitation: reliable response assessment per iRECIST could not be made in a substantial portion of patients (almost 80% of the patients with iUPD had no confirmation of subsequent response), as none of the included randomized clinical trials were actually conducted in accordance with iRECIST. In our study, by collecting each study result incorporating complete assessment per both RECIST 1.1 and iRECIST, we were able to achieve the pooled results fully reflecting accurate assessment per both RECIST 1.1 and iRECIST. The similarity of conclusion between our study and Mulkey et al.’s supports the robustness of the present findings. Also, despite the inclusion of studies with heterogeneous designs, the overall evidence was consistent across studies without heterogeneity, again highlighting the stability of the findings. This may help making a firm conclusion regarding the utility of iRECIST for evaluating ICI treatments in clinical trials and practice.

This study had limitations. First, the different designs and patient populations of the included studies may have led to potential bias. For example, non-small cell lung cancer was the most commonly investigated malignancy in this meta-analysis (7 out 11 studies), while the other types of tumors such as melanoma, breast cancer, and urothelial carcinoma have been evaluated in 1–3 studies. In addition, there was no study to include gastrointestinal or pancreatobiliary cancers. Further research is required to explore the differences in pseudoprogression between cancer types. However, the evidence was consistent, and no significant heterogeneity was found in most of the meta-analyses. Second, the number of included studies was relatively small. Further large-scale trials or prospective studies are necessary, and the results from currently ongoing clinical trials using iRECIST are highly anticipated. Third, although we reconstructed the IPD as accurately as possible according to Guyot et al. [27] for the RMST analysis, the generated data may not have been exactly the same as true IPD. However, the method was validated with high reproducibility and no systematic error.

## 4. Materials and Methods

### 4.1. Search Strategy

The standard guidelines of Preferred Reporting Items for Systematic Reviews and Meta-Analyses (PRISMA) were followed [28]. A comprehensive search of MEDLINE and EMBASE databases was performed to identify relevant studies published before July 16, 2020. The following search terms were used: (immunotherapy OR checkpoint OR check-point OR check OR PD1 OR PD-L1 OR CTLA4 OR ipilimumab OR nivolumab OR pembrolizumab OR atezolizumab OR avelumab OR durvalumab) AND iRECIST. The search was limited to English-language studies. A detailed search strategy is provided in Table S2. To expand the search, the bibliographies of articles were screened for potentially suitable articles.

### 4.2. Eligibility Criteria

The titles and abstracts of articles were screened to identify the potentially relevant articles. The complete text of selected articles was meticulously reviewed to determine their relevance. Two reviewers (H.J.P. and G.H.K.) independently selected articles using a standardized protocol with the established inclusion and exclusion criteria. Disagreements were resolved by discussion. Based on the PICOS (population, intervention, comparison, outcome, study design) approach [29], studies fulfilling the following criteria were included: (a) population: patients with malignant solid tumors; (b) intervention: treatment including ICIs; (c) comparisons: treatment response and outcome assessment using both RECIST 1.1 and iRECIST; (d) outcomes: treatment efficacy endpoints including response rate and survival including PFS; and (e) study design: RCTs and observational studies (prospective or retrospective). The exclusion criteria were: (a) case reports, reviews, editorials, letters, and conference proceedings; (b) studies not within the field of interest; (c) studies with insufficient response assessment data; and (d) studies with overlapping cohorts.

### 4.3. Data Extraction

Two reviewers (H.J.P. and G.H.K.) independently extracted data from the studies, and disagreements were resolved by discussion. The following data were extracted: (a) study characteristics including authors, year of publication, and study design; (b) demographic and clinical characteristics of the patients including sample size, type of cancer, and type of ICIs; (c) response-related endpoints based on RECIST 1.1 and iRECIST: ORR and iORR, and DCR and iDCR; and (d) data required to estimate PFS and iPFS as survival endpoints based on RECIST 1.1 and iRECIST, respectively [30,31]. In five studies [14,15,18,20,21], according to methods proposed by Guyot et al., individual patient data were reconstructed by extracting data from the Kaplan–Meier curves using digital software (WebPlotDizitizer) to estimate the time-dependent probability of PFS [27]. Definitions of each endpoint are detailed below.

### 4.4. Definition of Endpoints for Treatment Efficacy

Endpoints per RECIST 1.1 were defined according to the FDA of United States guidance entitled “Clinical Trial Endpoints for the Approval of Cancer Drugs and Biologics” [30]. Endpoints per iRECIST were defined according to the consensus guidelines of iRECIST working group, which are almost identical with those per RECIST 1.1 except for the determination of PD [9]. By iRECIST, patients with initial unconfirmed progressive disease (iUPD) were subsequently evaluated for confirmation of progression (iCPD). If a case shows initially progressive disease (PD on RECIST 1.1 and iUPD on iRECIST) followed by tumor shrinkage, it is reset as iSD, iPR, or iCR upon subsequent assessment based on iRECIST.

ORR or iORR, a direct measure of tumoricidal activity of treatment, was defined as the proportion of patients showing complete response (CR or iCR) or partial response (PR or iPR) per RECIST 1.1 or iRECIST, respectively. DCR or iDCR, an index to measure the tumoristatic effects of treatment [31], was defined as the proportion of patients showing complete response (CR or iCR), partial response (PR or iPR), or stable disease (SD or iSD) per RECIST 1.1 or iRECIST, respectively.

We calculated the incidence rate of discordance in the date when PD was assigned (hereafter referred to as PD date) between RECIST 1.1 and iRECIST as the proportion of patients who had iUPD and was subsequently reset as iCR, iPR, or iSD per iRECIST. PFS and iPFS were calculated from the date of treatment initiation to the PD date or death, whichever occurred earlier, per RECIST 1.1 and iRECIST. When analyzing iPFS, if iUPD or iCPD was determined at subsequent assessments after iUPD without being reset as iSD, iPR, or iCR, the earliest time point of iUPD was assigned as the PD date per iRECIST.

### 4.5. Quality Assessment

Two reviewers (H.J.P. and G.H.K.) independently reviewed the risk of bias and methodologic quality of the included studies. The Cochrane Risk of Bias 2.0 [32] for randomized clinical trials and the Newcastle-Ottawa Scale [33] for non-randomized studies was used. Any discrepancy was resolved by discussion.

### 4.6. Statistical Analysis for Meta-Analysis

Considering the characteristics of study subjects and the ICI treatment were not the same across the studies, we used random-effects model to generate the summary estimate of the magnitude of effect. The pooled estimates of ORR and iORR, and those of DCR and iDCR, were calculated by a random-effects model with an inverse-variance weighting model. The differences between ORR and iORR and between DCR and iDCR were evaluated using the meta-regression with a dichotomous moderator as response evaluation criteria (i.e., RECIST 1.1 and iRECIST) [34]. Also, the pooled incidence rate ratio of ORR and iORR and that of DCR and iDCR were obtained to evaluate the difference between those estimates by a random-effects model with an inverse-variance weighting model. If the 95% CIs of the pooled incidence ratio rate include 1.0, it indicates that there is no significant difference between ORR and iORR or between DCR and iDCR. To check the robustness of the study results, sensitivity analyses were performed for each subgroup classified according to the tumor type, drug type, study design (i.e., randomized and non-randomized, and prospective or retrospective), and prior systemic treatment using studies with available data.

The incidence rate of PD date discordance was also pooled by a random-effects model with an inverse-variance weighting model. To quantitatively compare PFS and iPFS, we used RMST, a statistically valid alternative to the hazard ratio which requires the proportional hazards assumption. However, the proportional hazards might not be always assumed in comparing the effect of ICI treatment and conventional treatment due to the delayed separation of survival curves or cross-over of survival curves. The “restricted mean” is a measure of average survival time from 0 to a specified time point, and the RMST is equivalent to the area under the survival curve up to a specific time point and a statistically valid measure of survival without any model assumptions [35,36,37]. RMST was calculated for PFS (RMST_PFS_) and iPFS (RMST_iPFS_) at the truncation time point defined as the maximum time that was shorter than or equal to the lesser of the longest time of follow-up. The pooled difference of RMST_PFS_ and RMST_iPFS_ with the 95% CI was estimated using the DerSimonian–Liard random-effects model [38]. A difference (i.e., RMST_iPFS_–RMST_PFS_) greater than 0 indicated longer iPFS than PFS.

Heterogeneity was evaluated using the I² statistics and the Cochran Q-test. I² > 50% or *p* < 0.10 of the Q-test suggests substantial heterogeneity [39]. Funnel plots were used for visual assessment of publication bias, and rank correlation tests were used to detect asymmetry [40]. *p* values were two-sided, with *p* < 0.05 considered as significant. All statistical analyses were performed using R version 4.0.2 (R Foundation for Statistical Computing, Vienna, Austria). All statistical analyses were performed using R version 4.0.2 (R Foundation for Statistical Computing, Vienna, Austria). The metafor package was used to obtain the pooled estimates. The survRM2 package was used to derive the RMST estimates according to RECIST 1.1 and iRECIST (i.e., RMST_PFS_ and RMST_iPFS_) from each study. Both packages are available from the CRAN website (https://cran.r-project.org/).

## 5. Conclusions

The application of iRECIST had no impact on response-related endpoints but had a minor impact on a survival endpoint, compared to RECIST 1.1. Such a modest benefit of iRECIST should be considered when we design a clinical trial for immune checkpoint inhibitors.

## Figures and Tables

**Table 1:** Characteristics of the studies included in the meta-analysis.

Study	Study Design	Tumor	Agent (s)	Prior Systemic Therapy (%)	Median Follow-Up (Months)	No. of Patients	Median PFS per RECIST 1.1 (Months)	Median iPFS per iRECIST (Months)	Median OS (Months)
Katz et al. (2018) [12]	Observational (Retrospective)	NSCLC	Anti-PD-1 monotherapy	100	NA	166	NA	NA	NA
Tazdait et al. (2018) [13]	Observational (Retrospective)	NSCLC	Anti-PD-1 or anti-PD-L1 monotherapy or anti-PD-1 combined with targeted agent	100	8.2	160	NA	NA	11.3
Amrane et al. (2019) [14]	Observational (Retrospective)	Melanoma	Anti-PD-1 or anti-CTLA-4 monotherapy	21.6	NA	37	Not reached	Not reached	36.6
Beer et al. (2019) [15]	Observational (Prospective)	NSCLC	Anti-PD-1 or anti-PD-L1 monotherapy	92.9, 1 line	12	42	10.6	10.5	20.0
Lai et al. (2019) [16]	Observational (Retrospective)	Multiple (NSCLC, Melanoma, HCC)	Anti-PD-1 or anti-CTLA-4 monotherapy or combination of anti-PD-1 and anti-CTLA-4	NA	NA	21	NA	NA	NA
Voorwerk et al. (2019) ^b^ [17]	RCT	Breast cancer	Anti-PD-1 monotherapy	100	19.9	66	1.9	1.9	NA
Liang et al. (2020) [18]	Observational (Retrospective)	NSCLC	Anti-PD-1 or anti-PD-L1 monotherapy or combination of anti-PD-1 and anti-PD-L1	83.7, 1–3 lines	NA	43	5.4	6.2	Not reached
Mulkey et al. (2020) ^b^ [19]	Pooled analysis of 14 RCTs	Multiple (melanoma, SCC, NSCLC, RCC, HNSCC)	Anti-PD-1 or anti-PD-L1 monotherapy	NA	NA	4751	3.9	4.2	NA
			Anti-CTLA-4 monotherapy	NA	NA	613 ^a^	NA	NA	NA
Won et al. (2020) [20]	Observational (Retrospective)	NSCLC	Anti-PD-1 or anti-PD-L1 monotherapy	NA	6.7	189	3.8	4.1	12.1
Shah et al. (2020) [21]	Phase II clinical trial	Breast cancer	Combination of anti-PD-1 and chemotherapy	73.7, 1–6 lines	NA	30	4.0	4.0	15.4
Fukuokaya et al. (2020) [22]	Observational (Retrospective)	Urothelial carcinoma	Anti-PD-1 monotherapy	100, 1–2 lines	8.2	91	NA	NA	Not reached

^a^ For 613 patients treated with anti-CTLA-4, only the overall respone rate was provided. ^b^ Median PFS was provided, but individual patient data were not obtainable. Abbreviations: CTLA-4, cytotoxic T-lymphocyte-associated antigen 4; HCC, hepatocellular carcinoma; HNSCC, head and neck squamous cell carcinomaNA, not available; NSCLC, non-small cell lung cancer; OS, overall survival; PD-1, programmed cell death protein 1; PD-L1, ligand of programmed cell death protein 1; PFS, progression-free survival; RCC, renal cell carcinoma; RCT, randomized controlled trial; SCC, squamous cell lung cancer.

**Table 2:** Sensitivity analyses according to tumor type, drug type, study design, and prior treatment.

Characteristics	No. of Study	Overall Response Rate (%)			Disease Control Rate (%)			PD Date Discordance Rate (%)
		Pooled ORR	Pooled iORR	*p* Value	Pooled DCR	Pooled DCR	*p* Value	Pooled Rate of Discordance
Tumor Type								
NSCNC	4	22.3 (17.7–26.9)	23.6 (18.8–28.3)	0.72	51.5 (45.1–57.8)	54.4 (46.6–62.3)	0.56	2.9 (1.3–4.4)
Breast cancer	2 *	17.8 (9.6–26.1)	17.8 (9.6–26.1)	>0.99	29.6 (8.3–51)	29.6 (8.3–51)	>0.99	2.9 (−5.1–11)
Others	4 *	51.7 (44.8–58.5)	29.8 (24.7–35)	>0.99	44.9 (33.3–56.6)	51.7 (44.8–58.5)	0.33	4.9 (4.3–5.5)
Drug type								
Anti-PD-1 or anti-PD-L1 monotherapy	6	24.8 (20–29.7)	26.4 (21–31.7)	0.68	45.3 (34.1–56.6)	48.9 (36.9–60.9)	0.67	4.7 (3.9–5.5)
Others	5	21.3 (14.8–27.7)	21.4 (15.3–27.5)	0.98	45.5 (31.9–59)	50.2 (42.8–57.5)	0.63	2.7 (0.8–4.7)
RCT vs Non-RCT								
RCT	2	23.7 (15.8–31.6)	24.2 (15.9–32.5)	0.93	38.0 (5.8–70.2)	39.0 (4.8–73.2)	0.97	4.9 (4.3–5.5)
Non-RCT	9	23.1 (18.6–27.6)	24.9 (19.3–30.5)	0.63	47.3 (40.2–54.3)	51.0 (47.1–54.9)	0.36	3.4 (2.1–4.6)
Patient recruitment								
Prospective	4	22.6 (16.3–28.9)	23.5 (16.6–30.4)	0.85	47.8 (29.2–66.5)	49.6 (29.4–69.8)	0.90	4.5 (2.7–6.2)
Retrospective	7	26.2 (18.9–29.5)	25.8 (19.7–32)	0.69	44.5 (38.2–50.9)	49.3 (45.2–53.4)	0.23	3.5 (2.1–4.9)
Prior treatment								
Prior systemic treatment in all patients	4	23.6 (17.8–29.5)	25.2 (16.2–34.2)	0.78	35.4 (20.6–50.2)	40.0 (21.7–58.2)	0.72	5.4 (1.6–9.2)
Others	7	23.5 (18.3–28.8)	24.6 (19.1–30)	0.79	50.7 (43.8–57.6)	53.8 (48.7–59)	0.48	3.6 (2–5.1)

Data are percentages with 95% confidence intervals in parentheses. iRECIST-based estimates are indicated as “i”. * One study reported results for triple-negative breast cancer and hormone receptor-positive breast cancer separately. Abbreviations: DCR, disease control rate; ORR, overall response rate; PD, progressive disease; RCT, randomized controlled trial.

## Data Availability

The authors confirm that the datasets analyzed during the current study are available from the corresponding author upon reasonable request.

## Supplementary Materials

The following are available online at https://www.mdpi.com/2072-6694/13/1/120/s1, Figure S1: Risk of bias quality assessment for one randomized controlled clinical trial, Figure S2: Forest plots showing the pooled estimates of ORR and iORR, Figure S3: Forest plots showing the pooled estimates of DCR and iDCR, Figure S4: Funnel plots for visual appraisal of literature bias, Table S1: The Newcastle-Ottawa scale quality assessment for non-randomized studies, Table S2: Detailed search queries.
