# Supplementary material for: Comparison of RECIST 1.1 and iRECIST in Patients Treated with Immune Checkpoint Inhibitors: A Systematic Review and Meta-Analysis
Source: Cancers (Basel). 2021 Jan 1;13(1):120. doi: 10.3390/cancers13010120 (PMC7795764; doi:10.3390/cancers13010120)
Supplement: Supplementary file 1 [file cancers-13-00120-s001.pdf]

Supplementary Materials

Comparison of RECIST 1.1 and iRECIST in patients treated with immune checkpoint inhibitors: A systematic review and meta-analysis

Index

Table S1. The Newcastle-Ottawa scale quality assessment for non-randomized studies

Table S2. Detailed search queries.

Figure S1. Risk of bias quality assessment for one randomized controlled clinical trial

Figure S2. Forest plots showing the pooled estimates of ORR and iORR

Figure S3. Forest plots showing the pooled estimates of DCR and iDCR

Figure S4. Funnel plots for visual appraisal of literature bias

Table S1. The Newcastle-Ottawa scale quality assessment for non-randomized studies

Study	Selection				Comparability	Outcome			Total[a]
	Representativeness of the exposed cohort	Selection of the non-exposed cohort	Ascertainment of exposure	Demonstration that outcome of interest was not present at the start of the study	Comparability on the basis of design and analysis	Ascertainment of outcome	Adequate follow-up	Adequacy of follow-up of cohorts	
Kats et al. (2018)	1	1	1	0	2	1	0	0	6
Tazdait et al. (2018)	1	1	1	0	2	1	0	1	7
Amrane et al. (2019)	1	1	1	0	2	1	0	1	7
Beer et al. (2019)	1	1	1	1	2	1	1	0	8
Lai et al. (2019)	1	1	1	0	2	1	0	1	7
Liang et al. (2020)	1	1	1	0	2	1	0	1	7
Won et al. (2020)	1	1	1	0	2	1	1	1	8
Shah et al. (2020)	1	1	1	1	2	1	0	1	8
Fukuokaya et al. (2020)	1	1	1	0	2	1	0	1	7

[a]Each study could be awarded a maximum of 9: a maximum of 2 for the item regarding comparability and a maximum of 1 for the other seven items.

Table S2. Detailed search queries

No.	Search queries for MEDLINE
#1	“iRECIST"[Text Word] OR "immune RECIST"[Text Word]
#2	"immunotherapy"[Title/Abstract] OR "checkpoint"[Title/Abstract] OR "check-point"[Title/Abstract] OR "check"[Title/Abstract] OR "PD-1"[Title/Abstract] OR "PD-L1"[Title/Abstract] OR "CTLA-4"[Title/Abstract] OR "ipilimumab"[Title/Abstract] OR "nivolumab"[Title/Abstract] OR "pembrolizumab"[Title/Abstract] OR "atezolizumab"[Title/Abstract] OR "avelumab"[Title/Abstract] OR "durvalumab"[Title/Abstract]
#3	#1 AND #2
#4	#3 AND 2017/01/01:3000/12/31[Date - Publication] AND English[Language]
No.	Search queries for EMBASE
#1	irecist OR 'immune-recist' OR 'immune recist'
#2	'immunotherapy'/exp OR 'immunotherapy' OR immunotherapy OR checkpoint OR 'check point' OR check OR 'pd1'/exp OR 'pd1' OR pd1 OR 'pd l1' OR 'ctla4'/exp OR 'ctla4' OR ctla4 OR 'ipilimumab'/exp OR 'ipilimumab' OR ipilimumab OR 'nivolumab'/exp OR 'nivolumab' OR nivolumab OR 'pembrolizumab'/exp OR 'pembrolizumab' OR pembrolizumab OR 'atezolizumab'/exp OR 'atezolizumab' OR atezolizumab OR 'avelumab'/exp OR 'avelumab' OR avelumab OR 'durvalumab'/exp OR 'durvalumab' OR durvalumab
#3	#1 AND #2
#4	#3 AND [2017-2020]/py AND [english]/lim

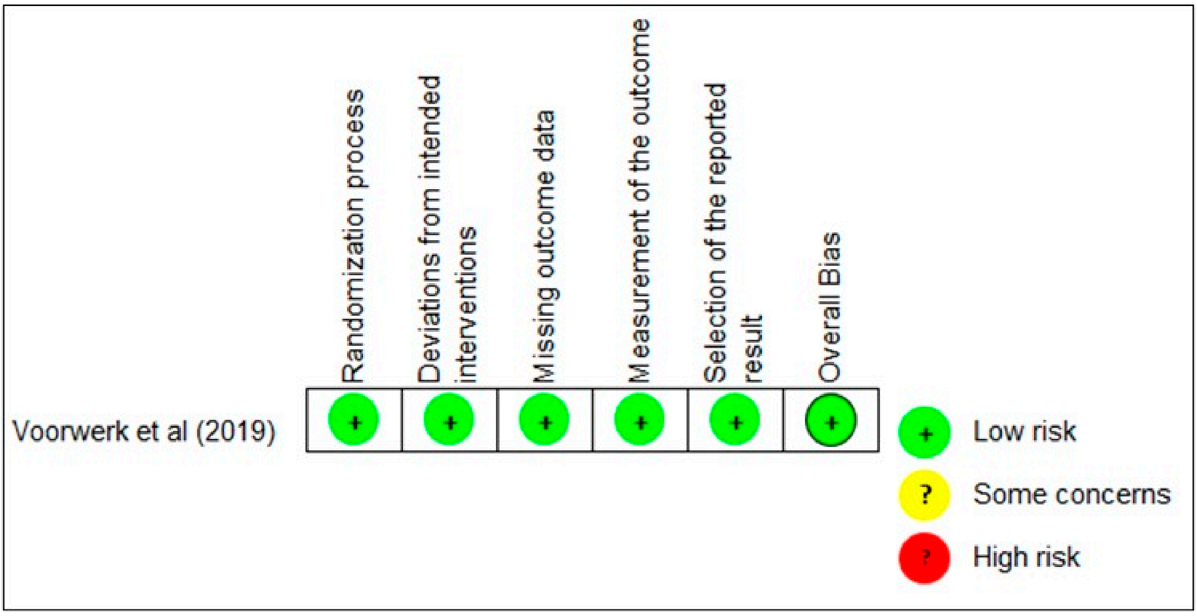

Figure S1. Risk of bias quality assessment for one randomized controlled clinical trial

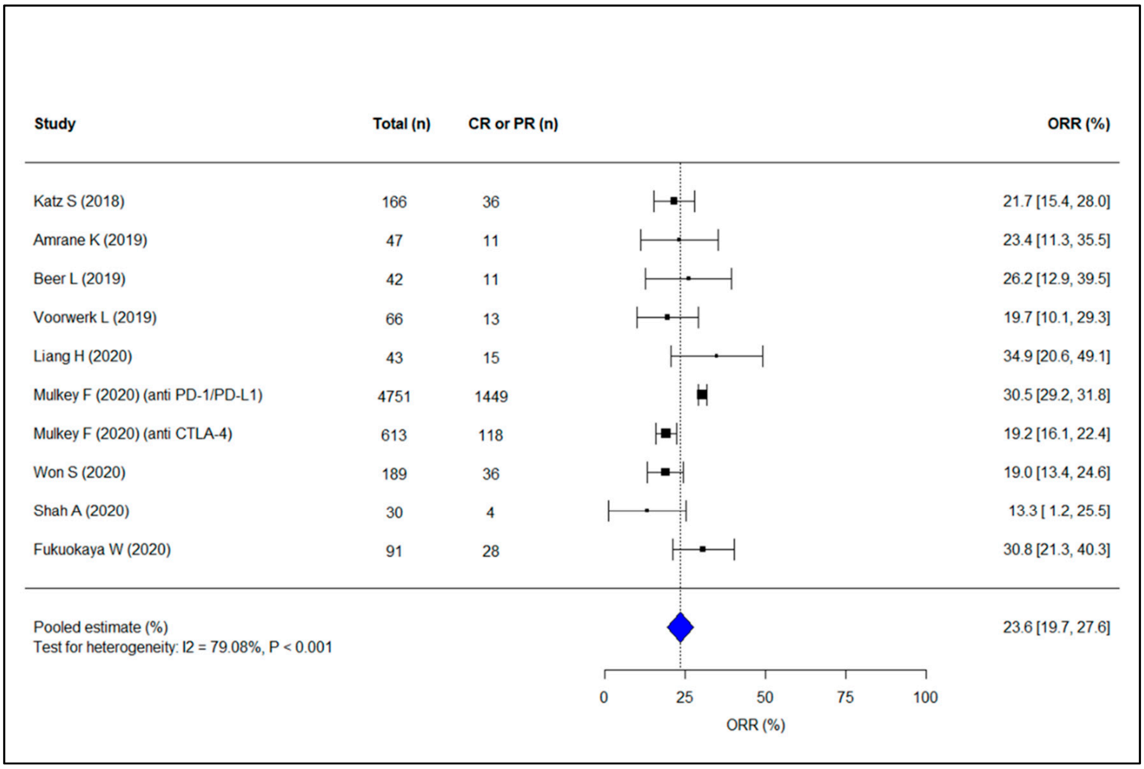

(A)

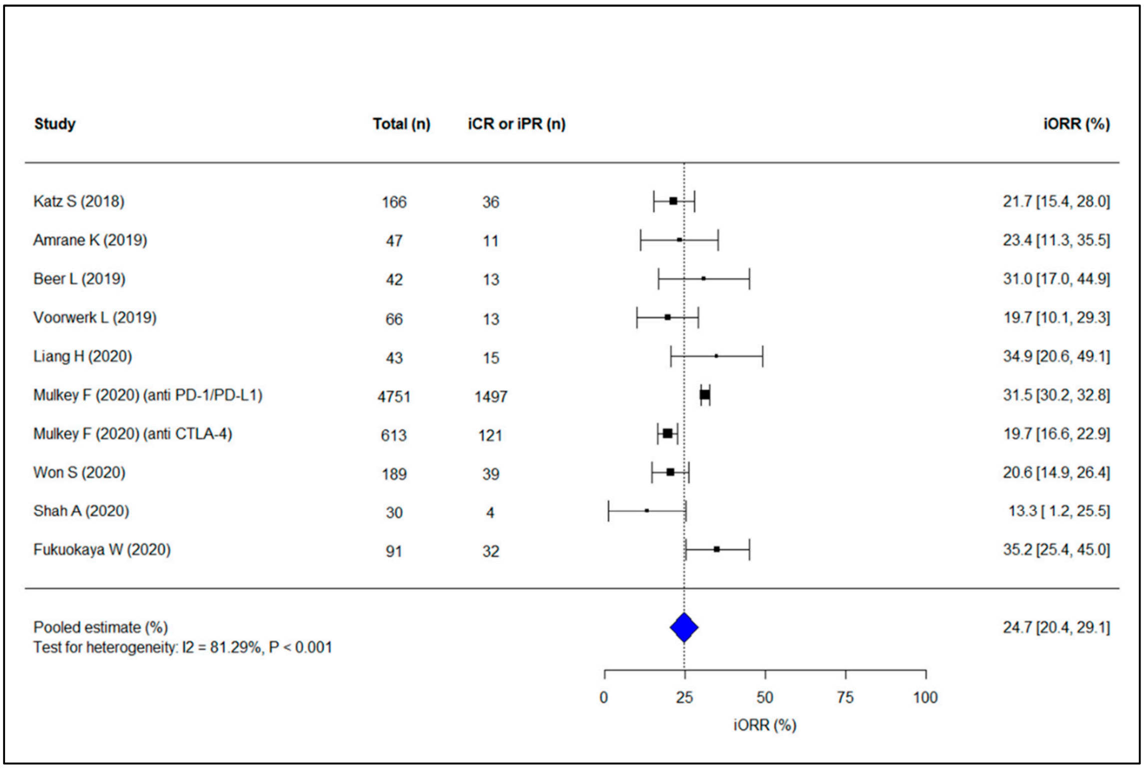

(B)

Figure S2. Forest plots showing the pooled estimates of (**A**) ORR and (**B**) iORR. The pooled ORR was 23.6% (95% CI, 19.7–27.6%), and the pooled iORR was 24.7% (95% CI, 20.4–29.1%). CI, confidence interval; ORR, overall response rate.

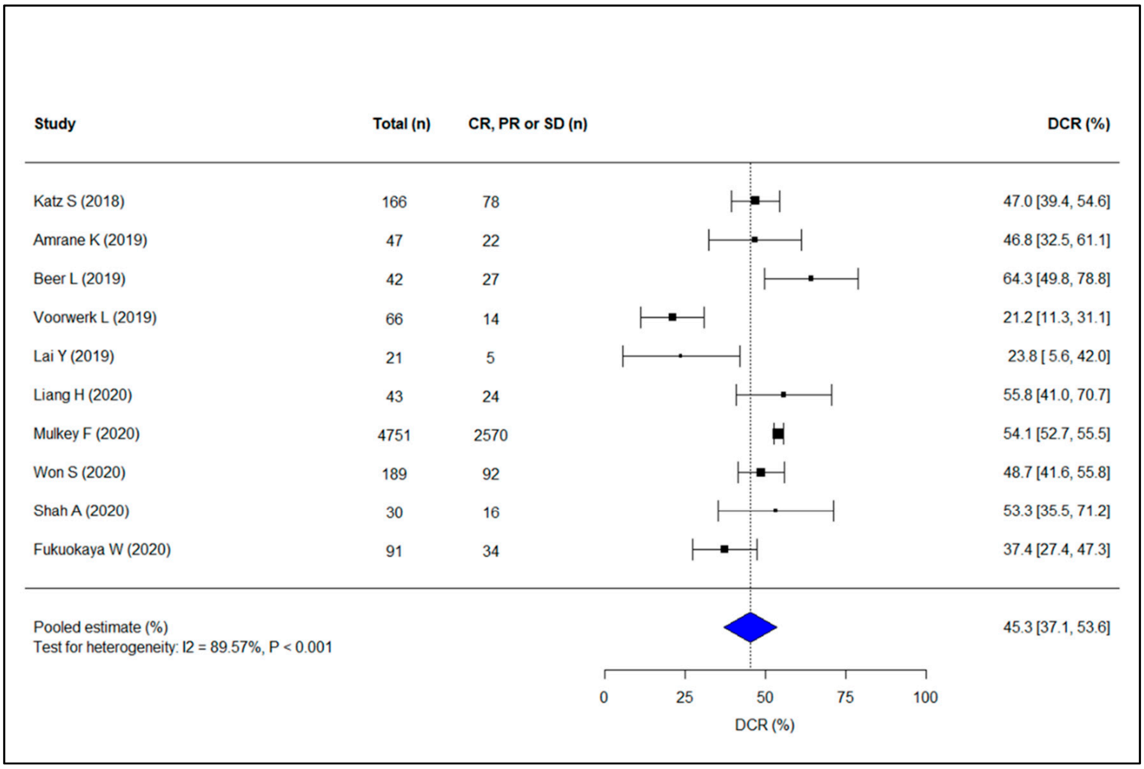

(A)

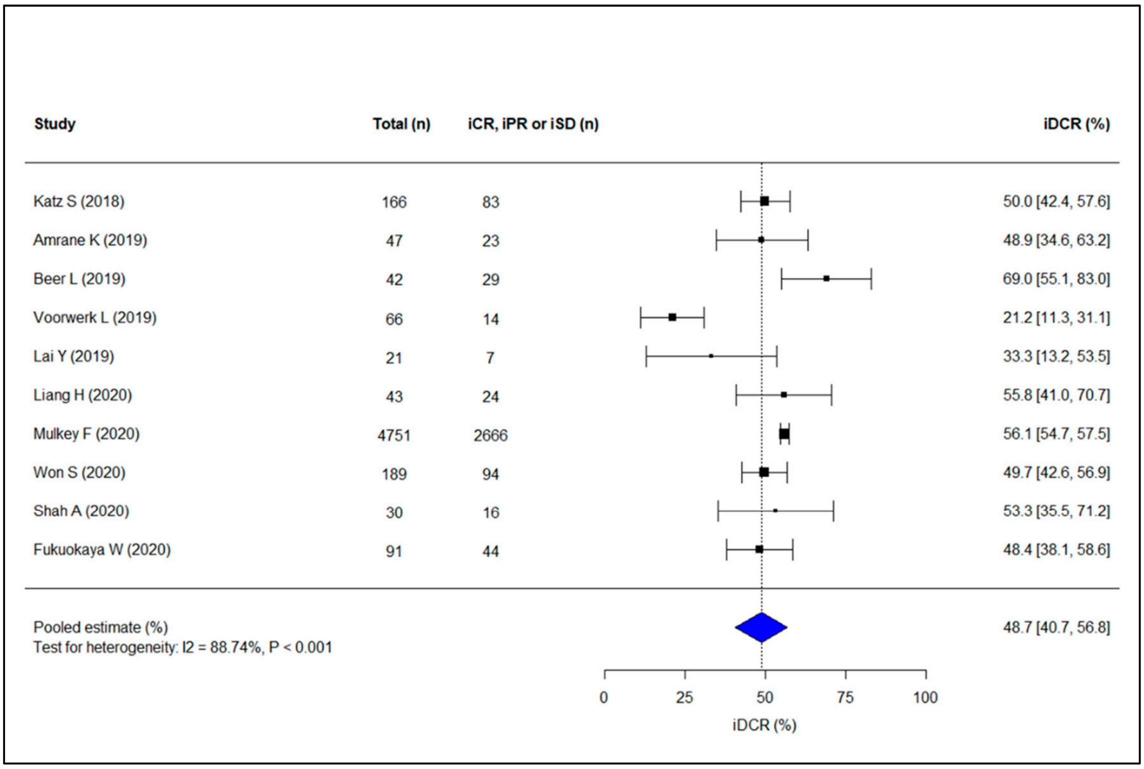

(B)

Figure S3. Forest plots showing the pooled estimates of (**A**) DCR and (**B**) iDCR. The pooled DCR was 45.3% (95% CI, 37.1–53.6%), and the pooled iDCR was 48.7% (95% CI, 40.7–56.8%). CI, confidence interval; DCR, disease control rate.

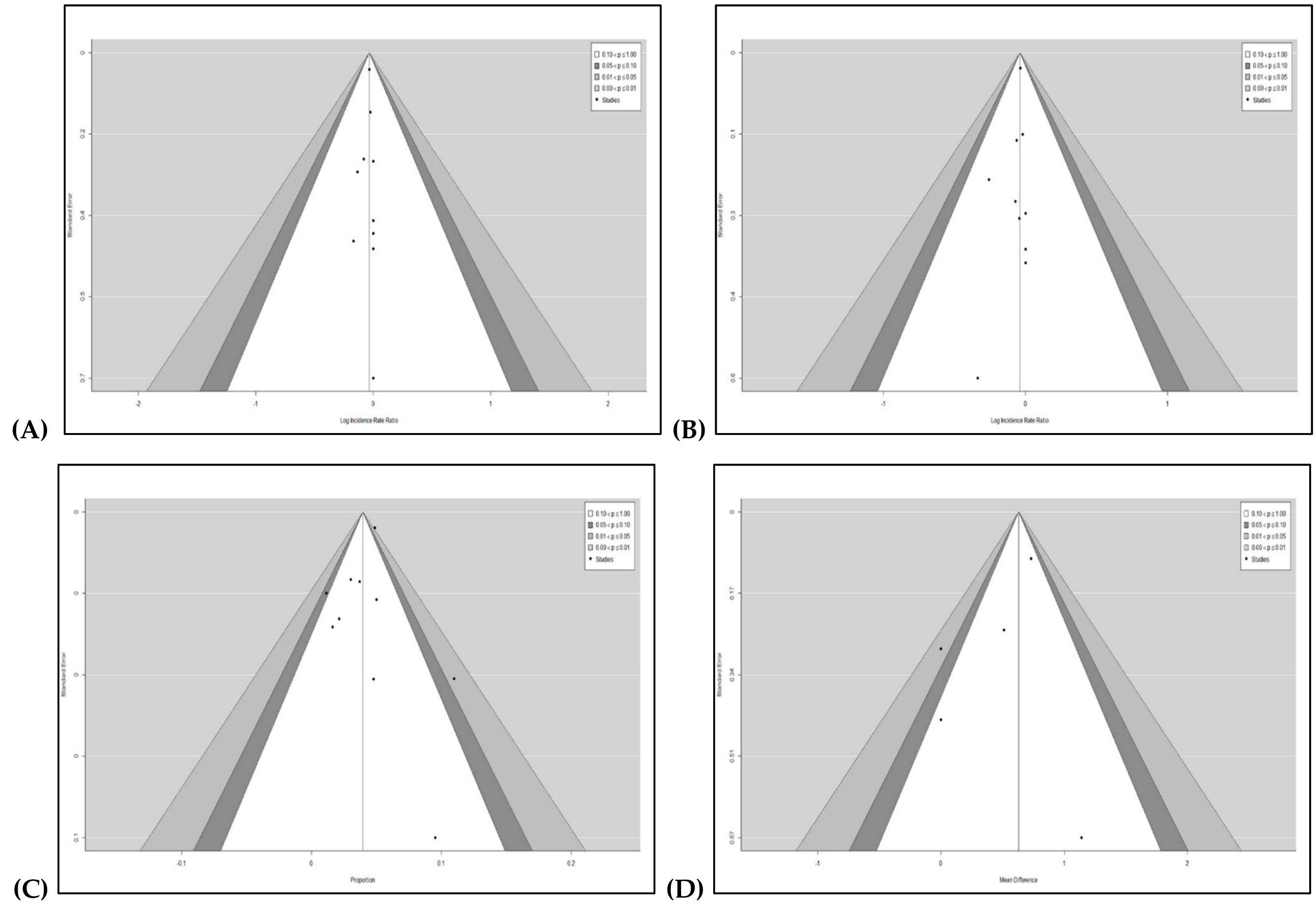

Figure S4. Funnel plots for visual appraisal of literature bias when pooling (**A**) IRR of ORR and iORR, (**B**) IRR of DCR and iDCR, (**C**) incidence rate of PD date discordance, (**D**) difference between RMST of PFS and iPFS. BOR, best overall response; DCR, disease control rate; IRR, incidence rate ratio; ORR, overall response rate; RMST, restricted mean square time.
